# Longitudinal relaxation rate (${R}_{1}$) in lung: a systematic review and meta-analysis

**DOI:** 10.1007/s10334-025-01259-x

**Published:** 2025-05-16

**Authors:** Lucy Edwards, Geoff J. M. Parker, John C. Waterton, Marta Tibiletti

**Affiliations:** 1 Bioxydyn Limited, St James Tower, 7 Charlotte Street, Manchester, M1 4DZ UK; 2 Department of Medical Physics and Biomedical Engineering, UCL Hawkes Institute, University College London, 1St Floor 90 High Holborn, London, WC1 V 6LJ UK; 3 Centre for Imaging Sciences, University of Manchester, Manchester, UK

**Keywords:** Magnetic resonance imaging, ${T}_{1}$Mapping, Lung imaging, Lung MRI

## Abstract

**Objective:**

The lung magnetic resonance longitudinal relaxation rate ${R}_{1}$ varies with disease but reported values in healthy subjects (HS) also differ markedly between studies.

**Objective:**

To evaluate the reported values and variances of observed ${R}_{1}$ accounting for field strength ${B}_{0}$, acquisition method, and disease.

**Materials and methods:**

A systematic literature search was performed to identify studies quantifying ${R}_{1}$ or its reciprocal ${T}_{1}$. The relationship between field strength and observed *R*_1_ was analysed in all healthy subject data. Data were fit to the heuristic equation ${R}_{1}=B* {(B}_{0 }^A)$. The variances between-study $bs$, within-study-between-subject $wsbs$, and within-study-within-subject $wsws$, in HS were assessed. Linear correlation between observed *R*_1_ and TE was also investigated.

**Results:**

Fifty-nine papers were selected, 50 quantifying *R*_1_ in HS and 20 in disease, representing 1559 human and non-human subjects, with ${B}_{0}$ ranging from 0.2 T to 9.4 T. In HS, the result of the fit on all healthy subjects was A = -0.227 ± 0.020 s^−1^ T^−1^, B = 0.923 ± 0.014 s^−1^ with variance components $bs>wsbs>wsws$. The reference values for *R*_1_ at ${B}_{0}$ between 0.55 T and 7 T were derived. For inversion recovery with gradient echo readout at 1.5 T, a previously observed negative relationship between ${R}_{1}$ and TE was confirmed.

**Discussion:**

We provide reference values for lung ${R}_{1}$ across all commonly used field strengths. The variation in HS lung observed ${R}_{1}$ reported in different studies in different centres likely reflects methodological differences. Investigators wishing to compare lung ${R}_{1}$ values with previous reports should take account of this irreproducibility, and multicentre projects should standardise to minimise between-centre variance.

**Supplementary Information:**

The online version contains supplementary material available at 10.1007/s10334-025-01259-x.

## Introduction

In the lung, the longitudinal water proton relaxation time ${T}_{1}$ (or better, the relaxation rate ${R}_{1}\equiv {T}_{1}^{-1}$) is sensitive to proportions of normal and inflamed parenchyma, as well as blood, mucus, and fibrosis. It is measurable on almost any MR scanner, does not require the use of injected or inhaled [1, 2] contrast agents, and has been proposed as a biomarker for assessing regional lung disease. Such a biomarker might be useful in several contexts [3]. In disease, previous studies have demonstrated altered ${R}_{1}$ in patients with chronic obstructive pulmonary disease (COPD) [4–7], emphysema [8], cystic fibrosis [6, 9] and interstitial pulmonary fibrosis (IPF) [10] relative to healthy control subjects. The potential uses in healthcare settings, in for example diagnostic, prognostic, predictive, or drug safety contexts, demand that the absolute value of the biomarker is consistent and reproducible between hospitals. This work assesses the consistency of lung ${R}_{1}$ across the published literature.

Numerous ${R}_{1}$ mapping methods have been proposed in the literature. Most accurate is the inversion recovery (IR) technique [11, 12], with saturation recovery (SR) an alternative: however, both require long repetition times (TR) resulting in slower data acquisition [11]. For faster acquisition, variations on these methods can be used, such as a fast spin echo or echo planar imaging readout [11]. Other rapid techniques include look-locker (LL) [13] and modified look locker inversion recovery (MOLLI), originally introduced for ${R}_{1}$ mapping of the heart [14]. Another, commonly used, rapid approach is the 3D variable flip angle (VFA) spoiled gradient echo method [15]. Although VFA is considered a convenient approach due to its speed, volume coverage, and ubiquitous availability, it is less accurate than other methods, particularly at higher field strengths, because of imperfect flip angles due to slab profile and transmit RF field inhomogeneity [16, 17].

Measuring ${R}_{1}$ in the lung poses significant challenges unique to this organ, chiefly the low tissue density and magnetic susceptibility difference at lung–air interfaces resulting in highly inhomogeneous local magnetic field gradients, which lead to a rapid dephasing in gradient echo imaging [18]. These are compounded by respiratory motion, which needs to be dealt with breath-holds, navigation pulses, gating, or registration, and by high levels of pulsatile blood flow, which can lead to motion and inflow-related errors. Moreover, these confounds themselves vary with disease.

The Solomon–Bloembergen equations predict that the ${R}_{1}$ of tissue decreases with magnetic field strength [19–21]: this has been confirmed in vivo inter alia in the brain [22], heart [23], musculoskeletal [24] and liver [25] and in the lung ex vivo [26]. The technical parameters need to also be considered, as observed ${R}_{1}$ has been reported to be dependent on echo time (TE), when measured using an IR multi-echo ultra-short TE (UTE) sequence [28].

The aim of this work was to systematically evaluate reported lung ${R}_{1}$ mean and variances with particular focus on the effects of field strength, acquisition method, and disease. This would permit future investigators to assess first, whether their measured ${R}_{1}$ and between-subject variances are consistent with previous work, and secondly any need for measurement harmonisation in multicentre studies.

Our work extends the work by Dietrich et al. [29]on healthy subjects at a single field strength (1.5 T), and by Bottomley et al. [26] who worked mainly ex vivo.

## Methods

This systematic review and meta-analysis followed the Preferred Reporting Items for Systematic Reviews and Meta-Analyses (PRISMA) guidelines [30]. The institutional review board approval was not required because all data were published previously.

### Literature search

A systematic literature search was performed in PubMed on 24 th October 2022 by one author to identify studies quantifying *R*_1_ in the lung. The following search terms were used (“lung”[All Fields] OR “pulmonary”[All Fields]) AND (“T1”[All Fields] OR “relaxation time” [All Fields] OR “longitudinal relaxation” [All Fields] OR “spin–lattice” [All Fields] OR “spin lattice”[All Fields]) AND (“MRI” [All Fields] OR “MR” [All Fields] OR “magnetic resonance”[All Fields]). There were no limits on date or language applied to the search. The search was repeated on 16 th May 2024 for papers published in the intervening interval.

One author screened the abstracts and titles of records identified in the search. The publications were included if either ${R}_{1}$ or ${T}_{1}$ was measured in the lung of human, swine, or rodent subjects of any age. There were no restrictions on method, study design or publication date. Both healthy and disease subjects were included. The same author screened the full-text articles for inclusion in the analysis. The publications were excluded if any of the following criteria were met: ${R}_{1}$ was measured but not reported; ${R}_{1}$ values reported only for neoplastic lesions; ${R}_{1}$ was measured ex-vivo; any language other than English or German; review articles. The citations and reference lists were also screened for additional publications to be included. We elected to work with ${R}_{1}$ rather than ${T}_{1}$ because, from a metrology perspective, ${R}_{1}$ is a ratio variable whilst ${T}_{1}$ is merely an interval variable [31].

### Data extraction and analysis

One author extracted data from each article for analysis. The mean $\mu$ and within-study-between-subject standard deviation ${\sigma }_{wsbs}$ of observed ${T}_{1}$ (s) or ${R}_{1}$ (s^−1^) in the lung were extracted. If the first (*q*_1_), median (*med*) and third (*q*_3_) quartiles were provided instead, the mean value $\mu$ and standard deviation ${\sigma }_{wsbs}$ were estimated as described by Wan et al. [32] as:

1 $$\mu =\frac{\left({q}_{1}+med+{q}_{3}\right)}{3} ; {\sigma }_{wsbs}=\frac{{q}_{3}-{q}_{1}}{1.35}$$

where the standard deviation was not reported, but a range was reported, ${\sigma }_{wsbs}$ was estimated using the range rule [25, 33]. To avoid bias, the data were manipulated in log space, with ${\mu }_{L}$ the estimated within-study-across-subject mean of $\text{ln}({R}_{1})$, and ${\sigma }_{L,wsbs}^{2}$ the estimated within-study-between-subject variance of $\text{ln}({R}_{1})$.

Our estimate of $${\mu }_{L}$$ was $$\text{ln}({R}_{1})$$ or $$-\text{ln}({T}_{1})$$, whilst the within-study-between-subject standard deviation in ln($${R}_{1})$$, $${\sigma }_{L,wsbs}$$, was estimated similarly to Waterton [25] as:

2 $${\sigma }_{L,wsbs}=0.5\cdot\text{ln}(\left({R}_{1}+{\sigma }_{wsbs}\right)/\left({R}_{1}-{\sigma }_{wsbs}\right))$$

If a different method was used to quantify observed ${R}_{1}$ (e.g. different field strength or TE) on the same subjects, each mean observed ${R}_{1}$ measurement was included as a separate value in the analysis. Where only individual data were reported, these were averaged to obtain one mean value per study. If the same experiment was performed on the same subjects as part of a repeatability study, the values were averaged to obtain one mean observed ${R}_{1}$ measurement. Such repeatability studies also provided an estimate of the within-study-within-subject (repeatability) variance in log space ${\sigma }_{L,wsws}^{2}$.

The region of interest (RoI) evaluated was also extracted. Typically, if reported, the mean ‘whole lung’ values were included in the analysis and taken to mean the average over left and right lungs. If only values for left and right lung regions were reported, these were averaged to estimate the mean and standard deviation for the ‘whole lung’. Where only ‘upper left lung’ or ‘upper right lung’ regions were reported, these were also included in the analysis.

Data regarding the study participants were also extracted. These included: species, number of subjects, smoking history, and their mean age and standard deviation, if available. The data were classified as either ‘healthy’ or ‘disease’ subjects. Control subjects who may have had other pathologies, but no known pathology in the lung, were also considered as healthy subjects. If available, the mean age and its standard deviation were extracted. If the median and IQR were reported in place of the mean, the mean age and standard deviation were estimated by Eq. 1. If only the range was reported, the mean age was estimated to be the arithmetic mean of the minimum and maximum age and the standard deviation was estimated by the range rule [33].

Other data regarding study methods and imaging parameters were extracted. These included field strength, TE, breathing method (breath-hold, breath-hold in inspiration or expiration, free breathing), ${T}_{1}$ mapping method (inversion recovery, variable flip angle, variable repetition time), and sequence. The data were classified by the ${T}_{1}$ mapping method used as either inversion-recovery (IR), variable-flip angle (VFA), look-locker (LL), modified look-locker inversion recovery (MOLLI), or mixed. The data were also categorised by the MR signal sampling used, either spin echo (SE), gradient echo (GRE), ultra-short echo time (UTE), zero echo time (ZTE), steady-state (SS) or mixed. If a range was given instead of a single value for TE, the arithmetic mean of the minimum and maximum values was used. Where a field strength of 3 T was reported for studies using a Siemens scanner, a value of 2.89 T was used in data analysis [34] and for Bruker scanners operating at 300 MHz or 400 MHz values of 7.05 T and 9.39 T respectively were used.

The relationship between field strength and observed ${R}_{1}$ was analysed in all healthy subject data. The data were fit for $A$ and $B$ by the heuristic:

3 $${R}_{1}=B*({B}_{0}^A)$$

weighted by ${\left({\sigma }_{L,wsbs}\right)}^{-2}$. Other fitting strategies, omitting non-human data, omitting gradient-echo readouts, or employing different weighting schemes, were also explored. In addition, a Bloembergen-type fit using parameters from Diakova et al. [35] was explored:

4 $$\begin{aligned}{\text{ln}(R}_{1})&=\text{ln}(C\cdot {\omega }_{0}^{-0.60}+D\cdot {\tau }_{D}\\&\quad\cdot \left[\text{ln}(\left(1+{\left({\tau }_{D}\cdot {\omega }_{0}\right)}^{-2}\right)+4\cdot \text{ln}(\left(1+{\left({2\cdot \tau }_{D}\cdot {\omega }_{0}\right)}^{-2}\right)\right]+{R}_{1,\infty })\end{aligned}$$

where: ${\omega }_{0}=2.68\times {10}^{9} {s}^{-1}\cdot {B}_{0}$ s^−1^; ${\tau }_{D}=1.47\times {10}^{-11}$ s, the estimated translational correlation time at 310 K; and ${R}_{1,\infty }$ is ${R}_{1}$ at infinite field (extreme narrowing). Normalised observed ${R}_{1}$ values, ${R}_{1\_corr}$, were also created using Eq. 3 levelled at 1.5 T.

Variability in ${\text{ln}(R}_{1})$ was compared by assessing three components of variance: the between-subjects (population) variance ${\sigma }_{L,wsbs}^{2}$; the between-studies (reproducibility) variance ${\sigma }_{L,bs}^{2}$; and the within-study repeatability variance ${\sigma }_{wsws}^{2}$ where data could be extracted from the publications.

Linear correlation between observed ${R}_{1}$ and TE was studied in the form ${R}_{1}=a* \text{TE + b}$ (Eq. 4), but only on observed ${R}_{1}$ acquired at 1.5 T, as the field dependence of ${R}_{2}^{*}$ is unknown, and only using IRdata, as there were insufficient data for other methods. MOLLI and LL methods were considered as IR for this analysis. The correlations between IR spin echo and between IR ‘gradient echo’ (GRE, UTE, ZTE) with TE were separately assessed.

## Results

### Literature search

**Fig. 1:**
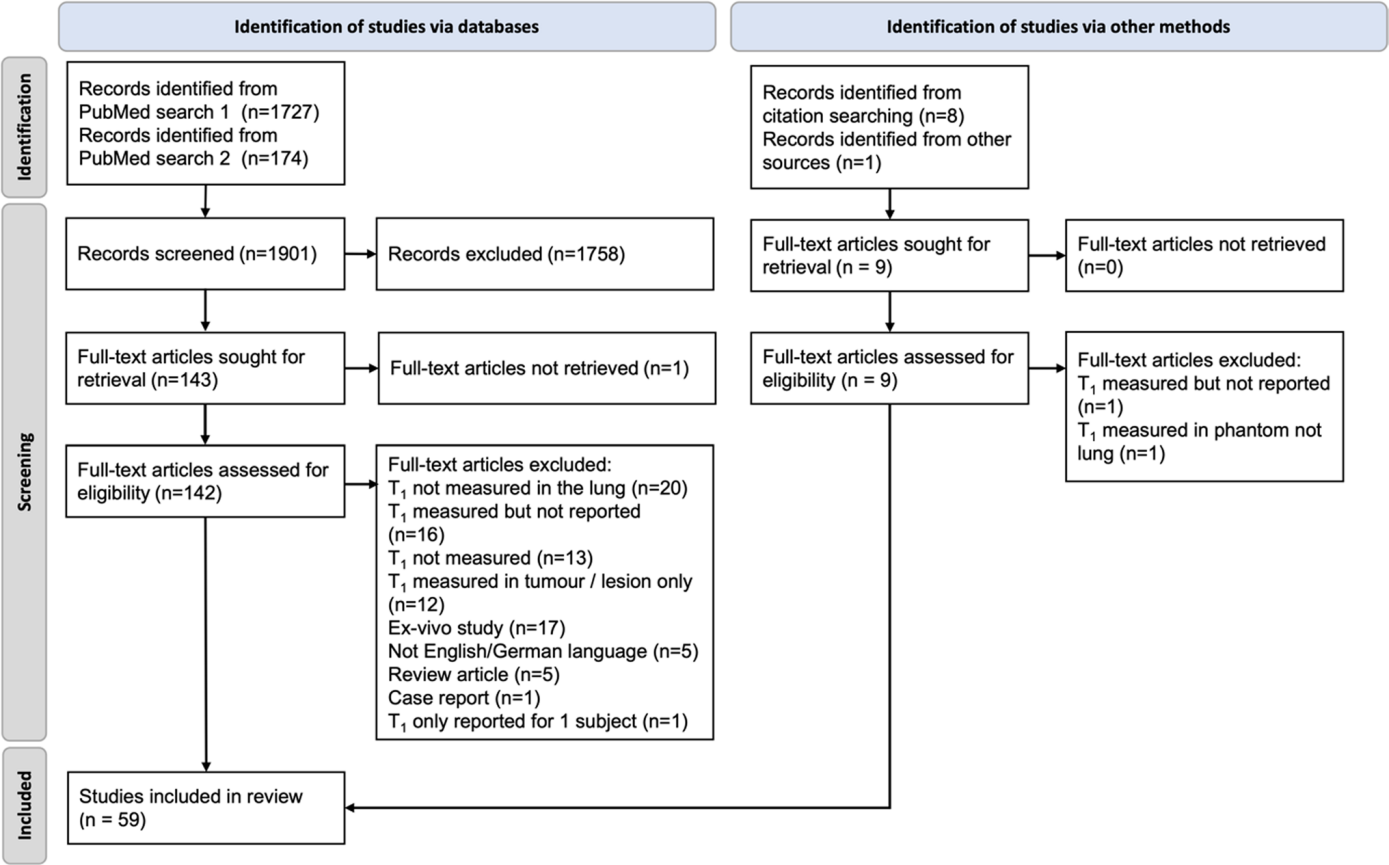
Preferred Reporting Items for Systematic Reviews and Meta-Analyses (PRISMA) flow diagram of data selection process

A PRISMA flow diagram of the study selection process is displayed in Fig. 1. The literature search retrieved 1727 results, of which 139 full text publications were selected for a detailed review. The second search retrieved a further 174 results, of which 4 full text publications were selected for a detailed review. Of these, 52 were selected for analysis and 6 additional publications were included after citation searching. One more publication was indicated by a study author. This resulted in a total of 59 publications for analysis with publication dates between 1986 and 2024. Of these, 50 quantified observed *R*_1_ in healthy subjects and 20 in disease subjects. These represented 1559 subjects (1450 humans, 65 mice, 30 rats and 14 swine), neglecting possible duplication. The number of subjects per study varied between 1 and 75. Some publications reportedmore than one method or multiple groups of subjects, in which caseeach mean observed ${R}_{1}$ value reported wascounted separately. This resulted in 115 mean observed ${R}_{1}$ values to be included in the analysis.

### Healthy subjects results

The study characteristics for healthy subjects can be found in Table 1. Sixty-five mean observed ${R}_{1}$ values were obtained from 50 publications in healthy subjects. These represented 484 humans, 57 mice, 15 rats and 14 swine.

**Table 1:** Summary of healthy subjects’ characteristics

Publication	Species	N	Age (y) mean (SD)	Smoking History	Field Strength (T)	TE (ms)	T_1_ mapping method	T_1_ mapping sequence	RoI evaluated
Edelman 1996 [53]	Human	6	24.5 (2.25)	n.a	1.5	25	IR	SE	Right/left lung
Chen 1998 [54]	Human	5	n.a	n.a	1.5	4.2	IR	SE	Upper right lung
Stock 1999 [55]	Human	8	39 (4.5)	Non-smokers	0.2/1.5	4.2	IR	W	Whole lung
Mai 1999a [56]	Human	3	33.5 (4.25)	n.a	1.5	38	IR	SE	Right/left lung
Mai 1999b [57]	Human	7	33.5 (4.25)	n.a	1.5	36	IR	SE	3 RoIs in posterior lung
Mai 2000[58]	Human	5	33.5 (4.25)	n.a	1.5	38	IR	SE	4 RoIs (2 each lung)
Löffler 2000 [59]	Human	9	28 (1)	n.a	1.5	4.2	IR	SE	10 RoIs in upper/middle/lower lung
Nakagawa 2001 [60]	Human	8	31 (3.5)	n.a	1.5	28.8	IR	SE	Upper, middle, lower peripheral lung fields—averaged
Hatabu 2001[61]	Human	9	24.5 (2.25)	n.a	1.5	25	IR	SE	Right/left lung
Jakob 2001 [62]	Human	6	28.5 (4.75)	n.a	1.5	1	IR	GRE	Right/left lung
Jakob 2002 [63]	Human	3	32 (4)	n.a	1.5	38	IR	SE	Upper right lung
Mai 2002a [64]	Human	6	33.7 (6.5)	n.a	1.5	21	IR	SE	Upper left lung
Mai 2002b [65]	Human	5	36.5(5.75)	n.a	1.5	20	IR	SE	Four RoIs, two on each lobe of the lung
Arnold 2004 [66]	Human	3	n.a	n.a	1.5	1.4	IR	GRE	Upper, middle, whole right lung/upper left lung
Stadler 2005 [7]	Human	10	29.7 (5.2)	n.a	1.5	1.4	LL	GRE	Whole lung
Naish 2005 [67]	Human	5	34.5 (2.25)	Non-smokers	1.5	16	SR	SE	Whole lung
Arnold 2007 [68]	Human	10	n.a	n.a	1.5	1.4	IR	GRE	Whole lung
Nichols 2008 [69]	Human	16	31 (11)	n.a	3	0.93	IR	SE	Whole lung
Molinari 2008 [70]	Human	23	25	n.a	1.5	0.5	IR	GRE	Right/left lung
Beer 2009 [71]	Human	5	n.a	n.a	0.2	3.7	IR	GRE	n.a
Togao 2011 [72]	Rat	5	0.15	n.a	3	0.1	VFA	UTE	4 RoIs – 2 per lung
Zurek 2014 [73]	Mouse	10	n.a	n.a	4.7	3.5	IR	SE	Whole lung
Triphan 2015a [28]	Human	12	28 (2.5)	Non-smokers	1.5	0.07/0.5/1.2/1.65/2.3	IR	UTE	Whole lung
Triphan 2015b [74]	Human	7	n.a	n.a	1.5	0.07	IR	UTE	Whole lung
Renne 2015a [75]	Human	12	28.5 (7.3)	Non-smokers	1.5	0.8	IR	GRE	Whole lung
Renne 2015b [41]	Human	4	43 (3.5)	Non-smokers pack year of less than 1 year & non-smokers for past 5 years	1.5	0.8	IR	GRE	Whole lung
Wurnig 2016 [76]	Mouse	6	n.a	n.a	4.7	none	IR	ZTE	Whole lung
Alamidi 2016 [4]	Human	12	63 (12)	Smokers – pack years 0.3 ± 1	1.5	3	IR	SE	Whole lung
Kindvall 2016 [27]	Human	30	45 (12.5)	Never smokers/0 pack years of tobacco use	1.5	0.67	LL	GRE	Whole lung
Zurek 2016 [47]	Mouse	9	0.15	n.a	4.7	0.5	IR	UTE	Whole lung
Gai 2017 [36]	Human	9	37.6 (10.8)	n.a	3	0.13/1.4	SR	UTE	Whole lung
Mirsardraee 2016 [45]	Human	7	68.8 (14.4)	No medical history of smoking	3	1	LL	SS	RoIs of normal lung tissue guided by HRCT scan
Tibiletti 2016 [77]	Rat	6	0.5	n.a	7	0.32	IR	UTE	Right/left lung
Kaireit 2017 [6]	Human	12	27.75 (9.4)	Non-smokers	1.5	0.8	IR	GRE	Whole lung
Kindvall 2017 [78]	Human	39	43 (10.5)	n.a	1.5	0.67	LL	GRE	Whole lung
Bauman 2017 [79]	Human	4	n.a	n.a	1.5	0.81	IR	SS	Whole lung
Guo 2018 [80]	Mouse	6	0.4	n.a	7	0.075	VFA	UTE	Whole lung
Alamidi 2018 [81]	Mouse	8	0.33	n.a	4.7	0.008	VFA	UTE	Whole lung
Bauman 2018 [38]	Human	7	39 (5)	n.a	1.5	0.71	IR	SS	Whole lung
Assländer 2018 [82]	Human	5	30 (2)	Non-smokers	3	0.63	IR	GRE	Whole lung
Kern 2020 [83]	Human	10	37.7 (11.1)	Non-smokers – pack years less than 1 year	1.5	0.66	IR	GRE	Whole lung
Campbell-Washburn 2019 [84]	Human	5	35 (14)	n.a	0.55	1.3	MOLLI	SS	Whole lung
Kuethe 2019 [49]	Rat	4	n.a	n.a	1.89	none	IR/VFA	SS/ZTE	Whole lung
Saunders 2020 [10]	Human	10	29.9 (5)	n.a	1.5	0.9	LL	GRE	Whole lung
Neemuchwala 2020 [9]	Human	5	11.2 (3.7)	n.a	1.5	1.63	MOLLI	SE	Whole lung
Cheriyan 2022 [46]	Human	17	61.2 (11.4)	Non-smokers	1.5	0.83	VFA	GRE	Whole lung
Tibiletti 2022 [37]	Human	9	49.4 (17.4)	n.a	1.5	0.4	IR	SE	Whole lung
Camastra 2022 [48]	Human	11	53 (22.4)	n.a	1.5	1.8	MOLLI	SS	Whole lung
Wieslander 2023 [85]	Human	10	26.7 (5.1)	n.a	0.55	1.28	MOLLI	SS	Whole lung
	Swine	14	n.a						
Kwiatkowski [86]	Mouse	18	0.23 (0.02)	n.a	9.4	0.26	VFA	UTE	Whole lung

*IR* inversion recovery, *VFA* variable flip angle, *LL* look-locker, *SR* saturation recovery, *MOLLI* modified look-locker inversion recovery, *UTE* ultra-short echo time, *TE* echo time, *RoI* region of interest, *SE* spin echo, *GRE* gradient echo, *ZTE* zero echo time, SS steady state, *n.a* not available

The RoI varied between whole lung measurements, left and right lung regions, and some studies evaluated smaller RoIs across one or both lungs.

The acquisition methods to quantify ${R}_{1}$ varied across the studies. Thirty-three studies employed an IR method, 5 LL, 5 VFA, 4 MOLLI, 2 SR, and 1 mixed. The ${R}_{1}$ mapping sequence varied across the studies, 18 applied an SE sequence, 15 GRE, 9 UTE, 6 SS, 1 ZTE and 1 mixed.

Nine field strengths were reported across all studies. The field strengths reported in rodent studies were 0.55–9.39 T and in humans 0.2–3.0 T. Of these, 33 measured ${R}_{1}$ at 1.5 T, 1 at 0.2 T and 1.5 T, 1 at 0.2 T, 2 at 0.55 T, 1 at 1.89 T, 2 at 2.89 T, 3 at 3.0 T, 4 at 4.7 T, and 2 at 7 T, 1 at 9.39 T.

**Fig. 2:**
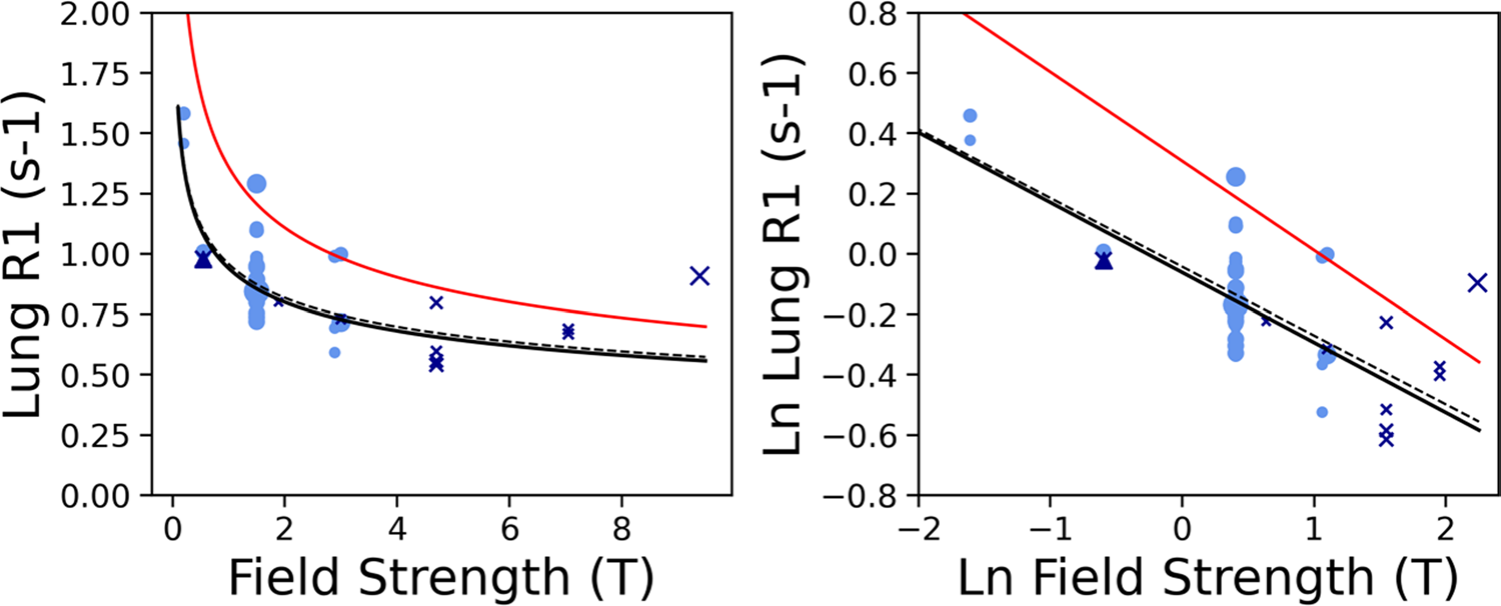
Representation of the dependence of observed *R*_1_ (s^−1^) on field strength. On the left the power relationship between the variables is shown, on the right the linear logarithmic relationship on the same data is shown. Only healthy human (cross marker, light blue) and healthy rodents (circle, dark blue) and swine (triangle, dark blue) are represented. Size of marker reflects number of subjects. Solid black line: result of the fit, weighted by 1/CoV for all studies. Dashed line: result of the fit, weighted by 1/CoV only for human studies

Figure 2 shows the fitted log–log relationship between observed ${R}_{1}$ and ${B}_{0}$ across the studies in healthy subjects.

The result of the Eq. 3 fit on all healthy subjects was A = − 0.227 ± 0.020 s^−1^ T^−1^, B = 0.923 ± 0.014 s^−1^.

Other fitting strategies gave similar results (supplementary material). The result of fitting Eq. 4 on all healthy subjects was: C = 9.170 ± 2.826 × 10^4^ s^−1^, D = 1.795 ± 1.546 × 10^8^ s^−1^, and *R*_1,∞_ = 0.618 ± 0.030 s^−1^.

Table 3 reports the predicted ${R}_{1}$ at different field strengths.

Data from the human studies provided an estimate of the between-subjects (population) variance ${\sigma }_{L,wsbs}^{2}$. This could be compared with the between-studies (reproducibility) variance ${\sigma }_{L,bs}^{2}$ and the within-study repeatability variance ${\sigma }_{L,wsws}^{2}$[4, 9, 36–38] (Table 4 and S2).

**Fig. 3:**
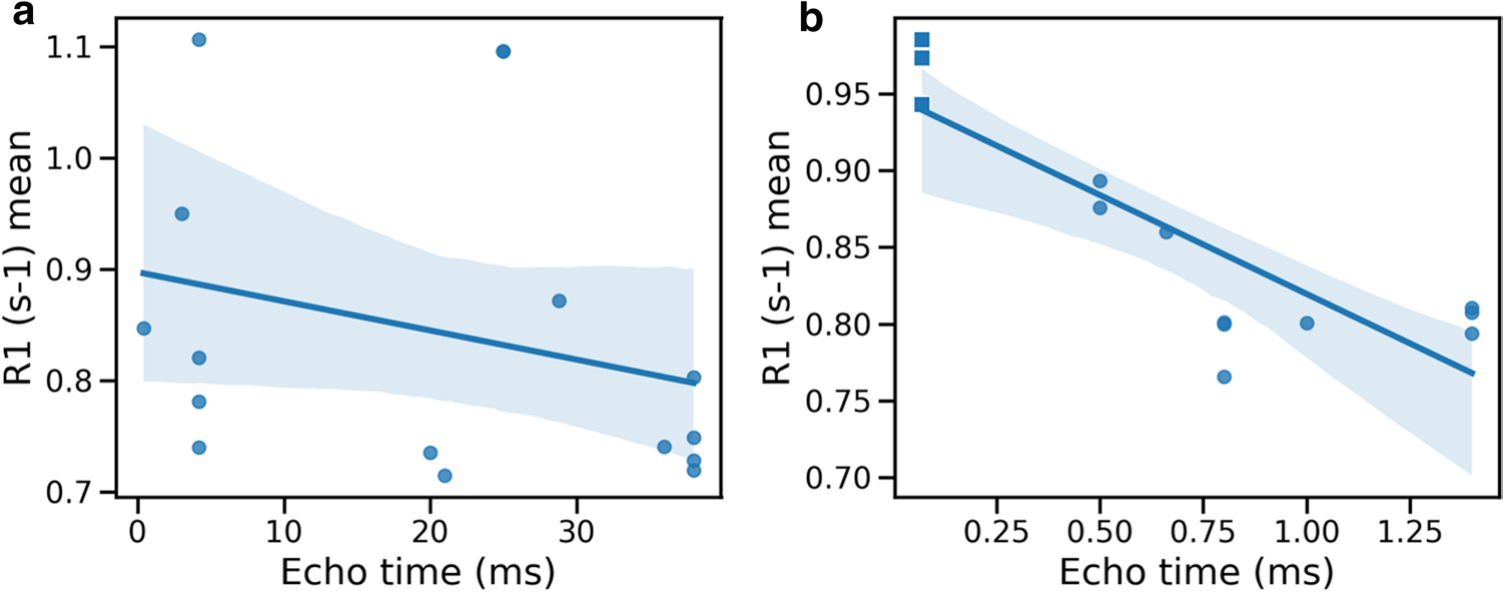
Scatterplots representing the correlations between the equivalent observed *R*_1_ at 1.5 T R_1_ (s^−1^) and echo time (TE, ms) in healthy subjects. **a** Studies employing the inversion recovery method and spin echo sequence (i.e. with refocussing of the transverse magnetisation) vs TE; **b** Studies employing the inversion recovery method and either gradient echo or ultra-short echo time (square markers). The straight line represents the best linear fit for each group and the shaded area is the 95% confidence area

No correlation was observed between observed ${R}_{1}$ and TE in studies employing IR methods and SE readout (Fig. 3a) (a = − 0.001 ± 0.0002 s^−1^/ms,, b = 0.859 ± 0.058 s^−1^, p = 0.62, see Eq. 4). A significant negative correlation was observed between observed ${R}_{1}$ and TE in studies employing IR methods and either, GRE, UTE or ZTE readouts (Fig. 3b) (a = − 0.095 ± 0.05 s^−1^/ms, b = 0.92 ± 0.22 s^−1^, p = 0.001).

### Disease subjects’ results

**Fig. 4:**
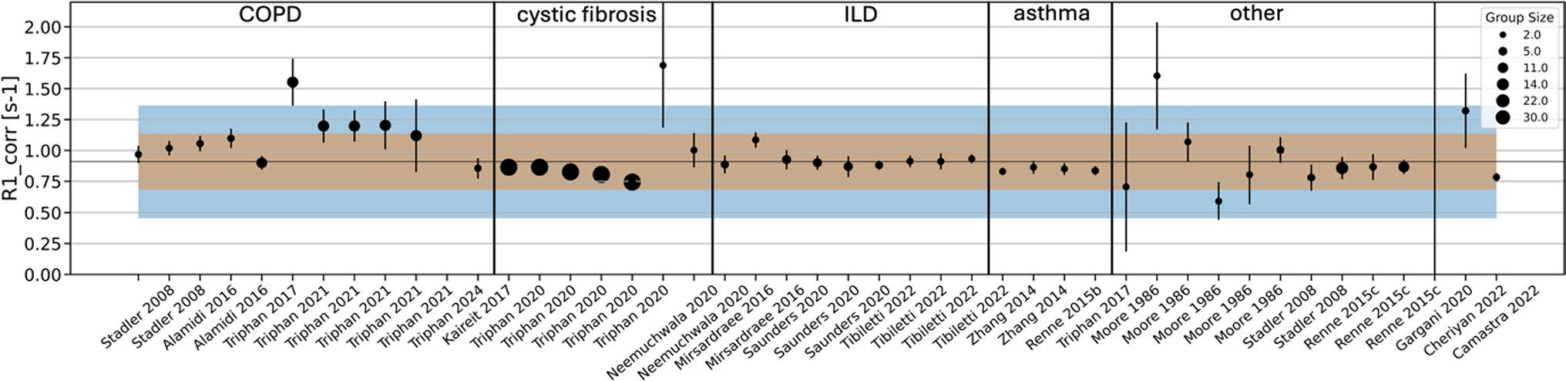
Mean *R*_1__corr (s^−1^) in the lung in human disease groups. Symbol size represents the group size; symbol error bar represents intra-study SD; horizontal black line is the weighted mean for healthy subjects across all studies; shading represents ± 1 and ± 2 SD of the weighted mean in healthy subjects. All values reported in each paper are shown

Data from diseased subjects were available from 20 publications. Some reported multiple methods to measure ${R}_{1}$ or studied several disease groups and therefore 50 mean observed ${R}_{1}$ values were included in this work. These represented 966 humans, 15 rats, and 8 mice. The study characteristics for disease subjects can be found in Table 2. Figure 4 presents all results from disease groups in human subjects, with weighted mean and weighted SD across all healthy subjects for reference.

**Table 2:** Summary of disease subjects’ characteristics

Publication	Species	N	Age(y) mean (SD)	Disease	Field Strength (T)	TE (ms)	T_1_ mapping method	T_1_ mapping sequence	RoI evaluated
Moore 1986 [50]	Human	3	n.a	Pulmonary oedema	0.35	28/56	VRT	SE	3 ROIs
		3		Alveolar proteinosis					
		3		Pneumocystis pneumonia					
		2		Lobar pneumonia					
		1		Acute radiation pneumonitis					
Stadler 2008 [8]	Human	11	58 (6)	Emphysema	1.5	1.4	LL	GRE	Whole lung
		14	62 (10)	Fibrosis					
Zhang 2015 [40]	Human	4	23 (5)	Mild asthma	1.5	3.2	IR	SE	Whole lung
		6	41 (12)	Severe asthma					
Renne 2015b [41]	Human	9	38 (5.5)	Asthma	1.5	0.8	IR	GRE	Whole lung
Renne 2015c [43]	Human	76	50	BOS	1.5	0.8	IR		Whole lung
Alamidi 2016 [4]	Human	12	66 (9)	COPD	1.5	3	IR	SE	Whole lung
		12	65 (6)	Severe COPD					
Zurek 2016 [47]	Mouse	8	0.15	PPE induced emphysema	4.7	0.5	IR	UTE	Whole lung
Mirsardraee 2016 [45]	Human	15	68.8 (14.4)	IPF	2.89	1	LL	SS	RoIs of normal/fibrotic tissue
Kaireit 2017 [6]	Human	12	14.7 (2.96)	CF	1.5	0.8	IR	GRE	Whole lung
Triphan 2017 [5]	Human	27	n.a	COPD	1.5	0.75	IR	GRE	12 RoIs- upper/middle/lower lung & anterior/ posterior
		12	n.a	Asthma					
Kuethe 2019 [49]	Rat	4	n.a	Atelectasis	1.89	none	IR/VFA	SS/ZTE	Whole lung
		4		LPS inflammation					
		3		VILI					
		4		Saline lavage oedema					
Gargani 2021 [44]	Human	30	46 (12.6)	Systemic sclerosis	1.5	1.1	IR	GRE	6 RoIs – upper/middle/lower lung
Triphan 2020 [42]	Human	75	8.6 (6.1)	CF	1.5	0.07/0.5/1.2/1.65/2.3	IR	UTE	Whole lung
Saunders 2020 [10]	Human	19	70.3 (7)	IPF	1.5	0.9	LL	GRE	Whole lung
Neemuchwala 2020 [9]	Human	4	11.4 (3.2)	CF	1.5	1.63	MOLLI	SE	Whole lung
		6	11 (5.3)	CF					
Triphan 2021 [39]	Human	30	67.7 (6.6)	COPD	1.5	0.07/0.5/1.2/1.65/2.3	IR	UTE	Whole lung
Cheriyan 2022 [46]	Human	12	67.8 (13.4)	Heart failure	1.5	0.83	VFA	GRE	Whole lung
Tibiletti 2022 [37]	Human	11	66.4 (9.6)	DIILD	1.5	0.4	IR	SE	Whole lung
		14	71.9 (7.18)	IPF					
		11	61.5 (12.5)	HP					
		5	58.5 (10.9)	CTD-ILD					
Camastra 2022 [48]	Human	11	55 (22.2)	COVID-19	1.5	1.8	MOLLI	SS	Whole lung
Triphan 2024 [87]	Human	22	68 (6.2)	COPD	1.5	0.7	IR	UTE	Normal lung regions & perfusion defect regions

*N.a* not available, *BOS* bronchiolitis obliterans syndrome, *CF* cystic fibrosis, *COPD* chronic obstructive pulmonary disease, *CTD*-*ILD* connective tissue disease related—interstitial lung disease, *DIILD* drug induced interstitial lung disease, *HP* hypersensitivity pneumonitis, *IPF* interstitial pulmonary fibrosis, *VILI* ventilator-induced lung injury, *IR* inversion recovery, *VFA* variable flip angle, *LL* look-locker, *SR* saturation recovery, *MOLLI* modified look-locker inversion recovery, *UTE* ultra-short echo time, *TE* echo time, *RoI* region of interest, *SE* spin echo, *GRE* gradient echo, SS steady state, *ZTE* zero echo time, *LPS* lipopolysaccharide‐induced inflammation, *PPE* porcine pancreatic elastase

The relaxation time was studied in diseased subjects with various diseases including: three in patients with COPD[4, 5, 39], two asthma [5, 40, 41], three cystic fibrosis [6, 9, 42], one bronchial obliterans syndrome [43], one systemic sclerosis [44], two interstitial pulmonary fibrosis [10, 45], one heart failure [46], one porcine pancreatic elastase (PPE) induced emphysema [47], one COVID-19 infection [48] and five in mixed disease groups [5, 8, 37, 49, 50].

## Discussion

A systematic review of observed *R*_1_/T_1_ measurement in in-vivo lung tissue is presented and reference values at various field strength between 0.2 T and 9.4 T derived. A strong dependency between observed *R*_1_ and echo time was found in gradient echo acquisition at 1.5 T, in accordance with existing literature [28].

${R}_{1}$ is an important biomarker which has been studied for decades and plays a crucial role in deriving other measurands and biomarkers, such as contrast agent concentration in DCE-MRI, arterial spin labelling, or pO_2_ in OE-MRI. The use of such biomarkers often rests on a platform of evidence from previous studies, implicitly assuming that data from different studies are comparable. This is particularly important for susceptibility, diagnostic, prognostic, predictive and safety biomarkers [51]. Therefore, the aim of this study was to evaluate the consistency between studies (reflecting the reproducibility) of observed ${R}_{1}$ as a lung biomarker.

The data are consistent with the heuristic linear relationship Eq. 3 between $\text{log}\left({R}_{1}\right)$ and $\text{log}\left({B}_{0}\right)$ over the ${B}_{0}$ range of interest in most human studies. The slope of the relationship is very similar to that reported by Bottomley et al. [26], although our curve, based on in vivo data, is offset from their curve, which was based mainly on ex vivo room temperature data. The fit of Eq. 4, which benefits from the rodent data, suggests a plausible asymptotic ${R}_{1,\infty }$ of 0.6 s^−1^, although given the small number of data points at certain field strengths this must be considered a weak, albeit surprisingly accurate and precise, finding. Healthy lung ${R}_{1}$ values expected at various commonly employed field strengths, obtained from these published data using Eq. 2 are given in Table 3. Equation 3 might not be safe to extrapolate above 3 T because of extreme narrowing (${R}_{1,\infty }$ plateau at infinite field) and where Eq. 4 might be preferable, or below 0.1 T because of the quadrupolar dips between 0.016 T and 0.065 T [52].

**Table 3:** Predicted lung R_1_ and T_1_ values at different field strengths as obtained by the regression represented in Fig. 2 and Eq. 3. The uncertainty represents ± σ_*wsbs*_

Field/T	Expected R_1_ in healthy lung/s^−1^	Expected T_1_ in healthy lung/ms
0.55	1.057 ± 0.143	946
1.5	0.842 ± 0.114	1188
2.89	0.725 ± 0.098	1379
3.0	0.719 ± 0.097	1390

Only five studies (56 subjects) were found where within-subject repeatability could be extracted. The reported variances were not consistent (Table S2), and we chose to exclude one small outlying dataset. Some inter-subject variation is to be expected in any clinical biomarker. In this survey, we found a between-subject within-study coefficient of variation ${\sigma }_{wsbs}/\mu$ of 14% in healthy subjects (Table 4). Of note, the biological variation was less than the methodological between-study variation but much greater than the repeatability variation.

**Table 4:** Log-space variance components in normal human lung

Component	Variance	Mean over
Reproducibility ${{\varvec{\sigma}}}_{{\varvec{L}},{\varvec{b}}{\varvec{s}}}^{2}$	0.0227	65 studies
Population ${{\varvec{\sigma}}}_{{\varvec{L}},{\varvec{w}}{\varvec{s}}{\varvec{b}}{\varvec{s}}}^{2}$	0.0136	442 HV in 62 studies
Repeatability ${{\varvec{\sigma}}}_{{\varvec{L}},{\varvec{w}}{\varvec{s}}{\varvec{w}}{\varvec{s}}}^{2}$	0.0010	51 HV in 4 studies

Our study also confirmed that the methodology used can impact ${R}_{1}$ measurements, particularly on gradient-echo based acquisitions. Because of the rapid transverse relaxation in lung, particularly at high field strength, there is rapid signal attenuation as TE increases. It is plausible that the signal remaining at longer TE arises disproportionately from certain tissue components, such as mucus, with short TE tissues (e.g. cellular or connective tissue) becoming invisible. The data imply that high-${R}_{1}$ components may be more evident at lower TE, so observed ${R}_{1}$ measurements measured using gradient echo readouts may be biased unless TE is very short.

In diseased subjects, observed *R*_1_ tended to be elevated in COPD and depressed in CF, consistent with previous findings [4, 6, 9]. However, it should be noted that COPD subjects tend to be older whilst CF subjects tend to be younger, which may bias this result.

In each voxel, the observed *R*_1_ is subject to uncertainty because of instrument noise. However, even in the absence of noise, observed *R*_1_ may be affected by physiological variables such as inflowing blood and magnetisation transfer, whilst multiple non-exchanging proton pools within the voxel could lead to multi-exponential decay. If those non-exchanging pools also exhibit different *T*_2_ or *T*_2_* relaxation, then pulse sequence with non-zero echo time may differentially suppress the contributions of the different pools, causing, as we observe, anomalous observations of *R*_1_ in long-echo-time protocols.

No phantom controls for all the confounding effects seen in vivo. However, a necessary (although not sufficient) condition for the validity of an *R*_1_ measurement method is that it accurately retrieves known *R*_1_ values from a valid traceable phantom. Few studies in this survey reported the use of a phantom, so their accuracy is difficult to assess. A realistic phantom of lung tissue would include multiple tissue compartments and realistic microscopic geometry, to mimic potential multiexponential relaxation and the impact of magnetic susceptibility differences. Such a realistic design is currently unachievable, but simpler phantoms can still provide important evidence of bias in relaxation time measurements.

Different investigators used different methods, some of which are well-known to be at high risk of methodologic confounds inter alia due to pulse imperfections, noise, and physiological motion. Also, *R*_1_ may differ regionally as different lobes have different blood volumes. A strong finding of this work is that the between-study variance considerably exceeds the within-study between-subjects variance, implying methodological irreproducibility and the need for standardisation and/or rigorous calibration. Investigators who wish to measure *T*_1_ or *R*_1_ should take reasonable steps to ensure accuracy and consistency with other work, and methods should be validated ideally using a traceable phantom.

## Supplementary Information

Below is the link to the electronic supplementary material.

Supplementary file1 (DOCX 406 KB)
