# Supplementary material for: Longitudinal relaxation rate (R1) in lung: a systematic review and meta-analysis
Source: MAGMA. 2025 May 16;38(5):747–60. doi: 10.1007/s10334-025-01259-x (PMC12497672; doi:10.1007/s10334-025-01259-x)
Supplement: Supplementary file 1 — Supplementary file1 (DOCX 406 KB) [file 10334_2025_1259_MOESM1_ESM.docx]

# Longitudinal relaxation rate ($R_{1}$) in lung: a systematic review and meta-analysis

Lucy Edwards^1^, Geoff JM Parker^1,2^, John C Waterton^1,3^, Marta Tibiletti^1^

1: Bioxydyn Limited, St James Tower, 7 Charlotte Street, MANCHESTER, M1 4DZ, UK

2: UCL Hawkes Institute, Department of Medical Physics and Biomedical Engineering, University College London, LONDON, UK

3: Centre for Imaging Sciences, University of Manchester, MANCHESTER, UK

Corresponding Author: Professor Geoff JM Parker, Hawkes Institute, Department of Medical Physics and Biomedical Engineering, University College London, 1st Floor 90 High Holborn, London, WC1V 6LJ; [geoff.parker@ucl.ac.uk](mailto:geoff.parker@ucl.ac.uk).

Journal: Magnetic Resonance Materials in Physics, Biology and Medicine

# Supplementary Material

# Supplementary Material 1: relationship between ${\ln(R}_{1})$ and ${\ln(B}_{0})$

Each study $i$, with $N\left( i \right)$ subjects, provided a mean $R_{1}$, $\mu\left( i \right)$ and within-study-between-subjects variance $\sigma_{wsbs}^{2}\left( i \right)$. As described in Methods, data were manipulated in log space on $\ln(R_{1})$ and its variance $\sigma_{L,wsbs}^{2}$.

The relationship between observed $R_{1}$ and $B_{0}$ was investigated as follows. Firstly, (a) measurements from diseased lung were excluded, and all remaining measurements fitted using nls in R to a heuristic

$R_{1}=B*(B_{0}^A).$ Eq. S1

weighted by the respective inverse variances $\sigma_{L,wsbs}^{-2}(i)$. $a$has dimensions $\ln\left( \text{s}^{-1} \right)\ln\left( \text{T}^{-1} \right)$ while $b$ has dimensions $\ln\left( \text{s}^{-1} \right).$ Confirmatory fits were made: (b) weighted by $N\left( i \right)$; (c) unweighted; (d)(e)(f) likewise but in human only; (g) human only, weighted by the inverse variances, omitting gradient echo readouts. Differences between the fits were small although our preferred fit (a) appeared marginally better. Fit parameters were compared with values from Bottomley et al (1984).

Fits to Eq.S1 are given in Table S1 and shown in Fig. S1. Eq.S1 was preferred over Eq.S2 by the Akaike Information Criterion (weighting by inverse log space variances in both cases). Eq2 results are: C = (9.170±2.826)×10^4^, D = (1.795±1.546)×10^8^, $R_{1,\infty}/s^{-1}$= 0.618±0.030

Table S1. Fits to Eq.S1

|  | A | B |
| --- | --- | --- |
| 1. weighted by inverse variances | -0.227±0.020 | 0.923±0.014 |
| 1. weighted by N | -0.160±0.031 | 0.942±0.017 |
| 1. unweighted | -0.192±0.029 | 0.940±0.015 |
| 1. weighted by inverse variances, human only | -0.274±0.025 | 0.940±0.010 |
| 1. weighted by N, human only | -0.228±0.045 | 0.958±0.017 |
| 1. unweighted, human only | -0.240±0.040 | 0.950±0.016 |
| 1. weighted by inverse variances, human only, excluding GRE | -0.220±0.066 | 0.930±0.021 |
| Recalculated from Bottomley et al | -0.296±0.141 | 1.384±0.085 |


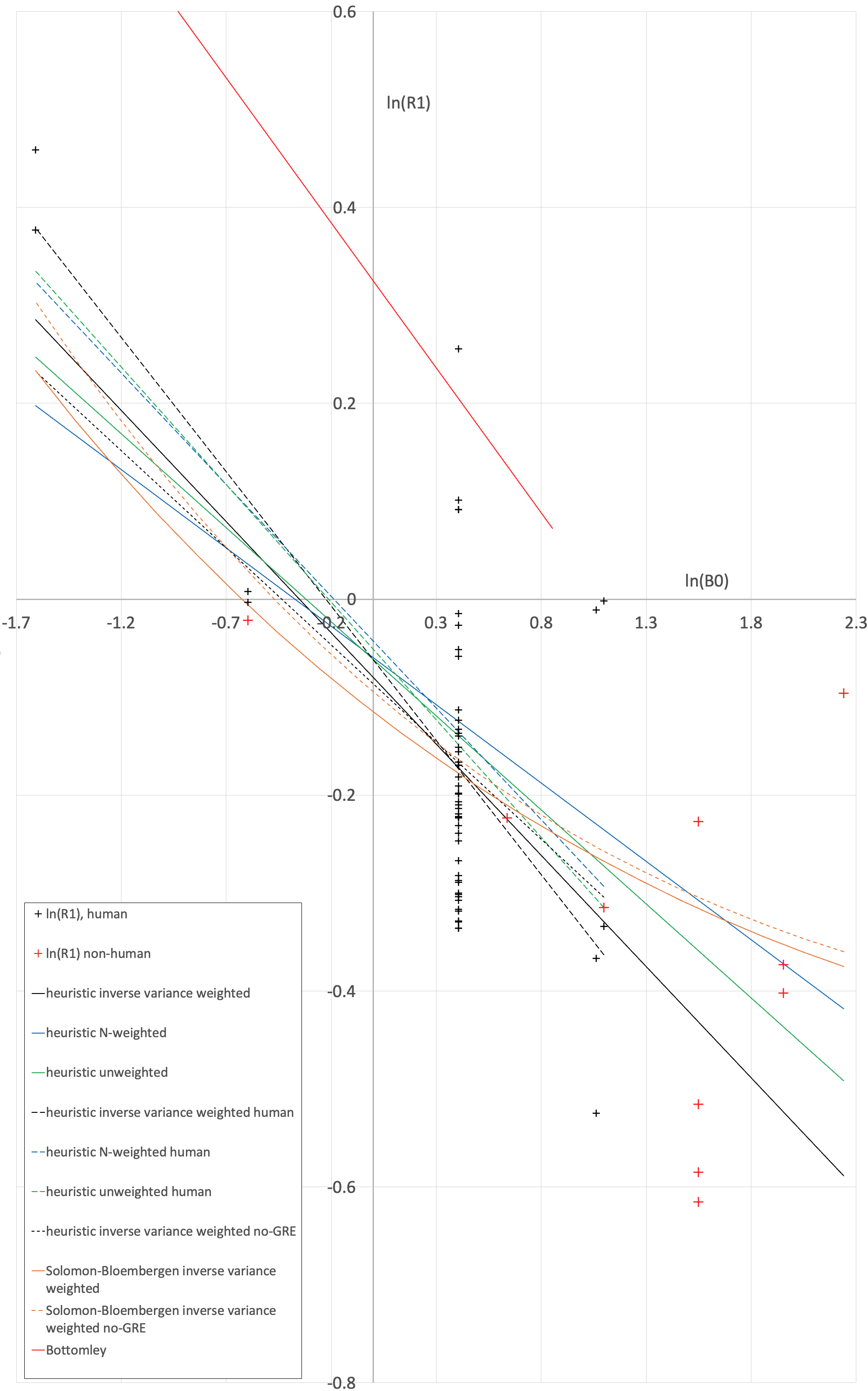


Fig. S1 Fits to Eq.S1 or Eq.S2

## Supplementary Material 2: Extract from R console

> R1lungs<-read.csv("LunglnR1Healthy.csv",header=TRUE)

> as.data.frame(R1lungs)

PMID R1 B0 wsbsSD lnR1 lnB0 unw unw_sp N_w N_sp_w var_ln_w var_ln_sp_w var_ln_sp_notGRE_w

1 26488310 0.949668 1.50 0.136189 -0.051643 0.405465 1 1 12 12 363.884 363.884 363.884

2 26558716 0.843882 1.50 0.040637 -0.169743 0.405465 1 1 30 30 0.000 0.000 0.000

3 18058926 0.715820 3.00 0.102450 -0.334327 1.098612 1 1 16 16 39.970 39.970 39.970

4 15906333 0.834028 1.50 0.041735 -0.181488 0.405465 1 1 10 10 102.361 102.361 0.000

5 15906333 0.750188 1.50 0.049738 -0.287432 0.405465 1 1 10 10 61.061 61.061 0.000

6 10373032 1.582278 0.20 0.000000 0.458866 -1.609438 1 1 8 8 134.317 134.317 134.317

7 10373032 1.106195 1.50 0.082168 0.100926 0.405465 1 1 8 8 80.725 80.725 80.725

8 11599066 0.871840 1.50 0.095483 -0.137150 0.405465 1 1 8 8 149.451 149.451 149.451

9 11418424 0.739754 1.50 0.122614 -0.301437 0.405465 1 1 5 5 694.418 694.418 694.418

10 35343008 1.290878 1.50 0.071157 0.255322 0.405465 1 1 17 17 56.962 56.962 0.000

11 27726231 0.998004 3.00 0.028059 -0.001998 1.098612 1 1 9 9 146.656 146.656 146.656

12 11274845 1.095890 1.50 0.170044 0.091567 0.405465 1 1 9 9 331.735 331.735 331.735

13 11747038 0.800480 1.50 0.060108 -0.222543 0.405465 1 1 6 6 989.187 989.187 0.000

14 29216201 0.801068 1.50 0.025443 -0.221809 0.405465 1 1 12 12 315.364 315.364 0.000

15 28494006 0.843977 1.50 0.045061 -0.169630 0.405465 1 1 30 30 516.532 516.532 0.000

16 27759935 0.727273 1.50 0.037111 -0.318454 0.405465 1 1 4 4 179.059 179.059 179.059

17 31729119 0.859796 1.50 0.054249 -0.151060 0.405465 1 1 10 10 265.681 265.681 0.000

18 10861881 0.820345 1.50 0.052683 -0.198031 0.405465 1 1 5 5 45.324 45.324 45.324

19 10861881 0.781250 1.50 0.120963 -0.246860 0.405465 1 1 4 4 224.106 224.106 224.106

20 26981454 0.989120 2.89 0.052110 -0.010940 1.061257 1 1 7 7 31.910 31.910 31.910

21 25604043 0.943396 1.50 0.035651 -0.058269 0.405465 1 1 12 12 699.585 699.585 699.585

22 25604043 0.813008 1.50 0.030448 -0.207014 0.405465 1 1 12 12 712.316 712.316 712.316

23 25604043 0.754148 1.50 0.028477 -0.282167 0.405465 1 1 12 12 700.645 700.645 700.645

24 25604043 0.738007 1.50 0.033291 -0.303801 0.405465 1 1 12 12 490.758 490.758 490.758

25 25604043 0.719942 1.50 0.027511 -0.328584 0.405465 1 1 12 12 684.170 684.170 684.170

26 24339056 0.800000 1.50 0.033209 -0.223144 0.405465 1 1 12 12 579.653 579.653 0.000

27 36252693 0.846883 1.50 0.068945 -0.166192 0.405465 1 1 9 9 150.218 150.218 150.218

28 10332866 0.719424 1.50 0.102953 -0.329304 0.405465 1 1 3 3 48.163 48.163 48.163

29 10862060 0.803213 1.50 0.060336 -0.219136 0.405465 1 1 5 5 176.553 176.553 176.553

30 10862060 0.749064 1.50 0.047887 -0.288931 0.405465 1 1 5 5 244.012 244.012 244.012

31 12112501 0.714796 1.50 0.067000 -0.335758 0.405465 1 1 6 6 113.152 113.152 113.152

32 12210943 0.735294 1.50 0.063176 -0.307485 0.405465 1 1 5 5 134.796 134.796 134.796

33 17685416 0.793651 1.50 0.040417 -0.231112 0.405465 1 1 10 10 384.933 384.933 0.000

34 16032679 0.787402 1.50 0.000000 -0.239017 0.405465 1 1 5 5 0.000 0.000 0.000

35 32557890 0.826446 1.50 0.096919 -0.190620 0.405465 1 1 10 10 72.045 72.045 0.000

36 32557890 0.869565 1.50 0.068472 -0.139762 0.405465 1 1 10 10 160.611 160.611 0.000

37 32557890 0.819672 1.50 0.067641 -0.198851 0.405465 1 1 8 8 146.180 146.180 0.000

38 31573398 0.997009 0.55 0.039461 -0.002996 -0.597837 1 1 5 5 94.037 94.037 94.037

39 28520198 0.714796 1.50 0.121900 -0.335758 0.405465 1 1 7 7 327.443 327.443 327.443

40 25816104 0.765501 1.50 0.019540 -0.267224 0.405465 1 1 4 4 38.767 38.767 0.000

41 15042464 0.810623 1.50 0.012186 -0.209952 0.405465 1 1 3 3 1720.396 1720.396 0.000

42 15042464 0.807563 1.50 0.043675 -0.213734 0.405465 1 1 3 3 4391.085 4391.085 0.000

43 12413560 0.728509 1.50 0.060230 -0.316755 0.405465 1 1 3 3 277.559 277.559 277.559

44 28419591 0.591716 2.89 0.000000 -0.524729 1.061257 1 1 5 5 8.112 8.112 0.000

45 28419591 0.693132 2.89 0.070171 -0.366535 1.061257 1 1 5 5 4.022 4.022 0.000

46 8898751 1.095890 1.50 0.063374 0.091567 0.405465 1 1 6 6 330.399 330.399 330.399

47 10195578 0.740741 1.50 0.078729 -0.300105 0.405465 1 1 7 7 0.000 0.000 0.000

48 18496048 0.893256 1.50 0.142179 -0.112882 0.405465 1 1 13 13 161.380 161.380 0.000

49 18496048 0.875657 1.50 0.139083 -0.132781 0.405465 1 1 10 10 190.248 190.248 0.000

50 32162080 0.883622 1.50 0.077688 -0.123726 0.405465 1 1 5 5 125.301 125.301 125.301

51 25044618 0.985383 1.50 0.173293 -0.014724 0.405465 1 1 7 7 47.365 47.365 47.365

52 25044618 0.973574 1.50 0.199621 -0.026781 0.405465 1 1 7 7 48.331 48.331 48.331

53 19657614 1.457726 0.20 0.319562 0.376878 -1.609438 1 1 5 5 1286.124 1286.124 0.000

54 36547479 0.855920 1.50 0.112288 -0.155578 0.405465 1 1 11 11 120.715 120.715 120.715

55 36822664 1.008065 0.55 0.082224 0.008032 -0.597837 1 1 10 10 582.739 582.739 582.739

56 26977928 0.557000 4.70 0.122474 -0.585190 1.547563 1 0 9 0 20.013 0.000 0.000

57 28812309 0.688468 7.05 0.023919 -0.373286 1.953028 1 0 6 0 276.073 0.000 0.000

58 26268414 0.597015 4.70 0.009353 -0.515813 1.547563 1 0 6 0 622.335 0.000 0.000

59 23878094 0.540541 4.70 0.040100 -0.615186 1.547563 1 0 10 0 3339.619 0.000 0.000

60 29517831 0.796813 4.70 0.041385 -0.227136 1.547563 1 0 8 0 394.167 0.000 0.000

61 38225796 0.908265 9.39 0.051309 -0.096219 2.239645 1 0 18 0 312.685 0.000 0.000

62 21761465 0.729927 3.00 0.017929 -0.314811 1.098612 1 0 5 0 71.587 0.000 0.000

63 30417929 0.800000 1.89 0.085871 -0.223144 0.636577 1 0 4 0 1990.318 0.000 0.000

64 27809405 0.668896 7.05 0.014548 -0.402126 1.953028 1 0 6 0 2113.334 0.000 0.000

65 36822664 0.978474 0.55 0.047021 -0.021761 -0.597837 1 0 14 0 432.356 0.000 0.000

> lnR1<-R1lungs[,5]

> lnB0<-R1lungs[,6]

> unw<-R1lungs[,7]

> unw_sp<-R1lungs[,8]

> N_w<-R1lungs[,9]

> N_sp_w<-R1lungs[,10]

> var_ln_w<-R1lungs[,11]

> var_ln_sp_w<-R1lungs[,12]

> var_ln_sp_notGRE_w<-R1lungs[,13]

> model2 <- nls(lnR1 ~ log2((R1A*(B0*2675221874.4)^(-0.6))+(R1B*1.4274e-11*(log2(1+(1.4274e-11*B0*2675221874.4)^(- 2)) + 4*log2(1+(2*1.4274e-11*B0*2675221874.4)^(-2))))+R1C), data=R1lungs, start=list(R1A=1e5, R1B=1e9, R1C=0.2), weights=var_ln_w, control=list(maxiter = 500, tol = 0.00001, minFactor = 1e-7, printEval = TRUE, warnOnly = FALSE))

It. 1, fac= 1, eval (no.,total): ( 1, 1): new dev = 458.61

It. 2, fac= 1, eval (no.,total): ( 1, 2): new dev = 401.411

It. 3, fac= 1, eval (no.,total): ( 1, 3): new dev = 401.395

It. 4, fac= 1, eval (no.,total): ( 1, 4): new dev = 401.395

> temp<-summary(model2,100); print(temp)

Formula: lnR1 ~ log2((R1A * (B0 * 2675221874.4)^(-0.6)) + (R1B * 1.4274e-11 *

(log2(1 + (1.4274e-11 * B0 * 2675221874.4)^(-2)) + 4 * log2(1 +

(2 * 1.4274e-11 * B0 * 2675221874.4)^(-2)))) + R1C)

Parameters:

Estimate Std. Error t value Pr(>|t|)

R1A 9.170e+04 2.826e+04 3.245 0.00194 **

R1B 1.795e+08 1.546e+08 1.161 0.25045

R1C 6.183e-01 2.976e-02 20.774 < 2e-16 ***

---

Signif. codes: 0 ‘***’ 0.001 ‘**’ 0.01 ‘*’ 0.05 ‘.’ 0.1 ‘ ’ 1

Residual standard error: 2.608 on 59 degrees of freedom

Correlation of Parameter Estimates:

R1A R1B

R1B -0.92

R1C 0.48 -0.76

Number of iterations to convergence: 4

Achieved convergence tolerance: 8.952e-07

> model1a <- nls(lnR1 ~ (R1AA*lnB0+log2(R1BB)), data=R1lungs, start=list(R1AA=-0.2, R1BB=1), weights=var_ln_w, control=list(maxiter = 500, tol = 0.001, minFactor = 1e-7, printEval = TRUE, warnOnly = FALSE))

It. 1, fac= 1, eval (no.,total): ( 1, 1): new dev = 449.557

It. 2, fac= 1, eval (no.,total): ( 1, 2): new dev = 449.175

> temp<-summary(model1a,100); print(temp)

Formula: lnR1 ~ (R1AA * lnB0 + log2(R1BB))

Parameters:

Estimate Std. Error t value Pr(>|t|)

R1AA -0.22662 0.02016 -11.24 <2e-16 ***

R1BB 0.93338 0.01299 71.84 <2e-16 ***

---

Signif. codes: 0 ‘***’ 0.001 ‘**’ 0.01 ‘*’ 0.05 ‘.’ 0.1 ‘ ’ 1

Residual standard error: 2.736 on 60 degrees of freedom

Correlation of Parameter Estimates:

R1AA

R1BB -0.61

Number of iterations to convergence: 2

Achieved convergence tolerance: 3.626e-05

> model1b <- nls(lnR1 ~ (R1AA*lnB0+log2(R1BB)), data=R1lungs, start=list(R1AA=-0.2, R1BB=1), weights=N_w, control=list(maxiter = 500, tol = 0.001, minFactor = 1e-7, printEval = TRUE, warnOnly = FALSE))

It. 1, fac= 1, eval (no.,total): ( 1, 1): new dev = 13.7466

It. 2, fac= 1, eval (no.,total): ( 1, 2): new dev = 13.7424

> temp<-summary(model1b,100); print(temp)

Formula: lnR1 ~ (R1AA * lnB0 + log2(R1BB))

Parameters:

Estimate Std. Error t value Pr(>|t|)

R1AA -0.16018 0.03090 -5.183 2.45e-06 ***

R1BB 0.94177 0.01656 56.865 < 2e-16 ***

---

Signif. codes: 0 ‘***’ 0.001 ‘**’ 0.01 ‘*’ 0.05 ‘.’ 0.1 ‘ ’ 1

Residual standard error: 0.467 on 63 degrees of freedom

Correlation of Parameter Estimates:

R1AA

R1BB -0.64

Number of iterations to convergence: 2

Achieved convergence tolerance: 1.632e-05

> model1c <- nls(lnR1 ~ (R1AA*lnB0+log2(R1BB)), data=R1lungs, start=list(R1AA=-0.2, R1BB=1), weights=unw, control=list(maxiter = 500, tol = 0.001, minFactor = 1e-7, printEval = TRUE, warnOnly = FALSE))

It. 1, fac= 1, eval (no.,total): ( 1, 1): new dev = 1.41733

It. 2, fac= 1, eval (no.,total): ( 1, 2): new dev = 1.41679

> temp<-summary(model1c,100); print(temp)

Formula: lnR1 ~ (R1AA * lnB0 + log2(R1BB))

Parameters:

Estimate Std. Error t value Pr(>|t|)

R1AA -0.19169 0.02908 -6.592 1.02e-08 ***

R1BB 0.93996 0.01549 60.688 < 2e-16 ***

---

Signif. codes: 0 ‘***’ 0.001 ‘**’ 0.01 ‘*’ 0.05 ‘.’ 0.1 ‘ ’ 1

Residual standard error: 0.15 on 63 degrees of freedom

Correlation of Parameter Estimates:

R1AA

R1BB -0.62

Number of iterations to convergence: 2

Achieved convergence tolerance: 1.951e-05

> model1d <- nls(lnR1 ~ (R1AA*lnB0+log2(R1BB)), data=R1lungs, start=list(R1AA=-0.2, R1BB=1), weights=var_ln_sp_w, control=list(maxiter = 500, tol = 0.001, minFactor = 1e-7, printEval = TRUE, warnOnly = FALSE))

It. 1, fac= 1, eval (no.,total): ( 1, 1): new dev = 189.215

It. 2, fac= 1, eval (no.,total): ( 1, 2): new dev = 189.052

> temp<-summary(model1d,100); print(temp)

Formula: lnR1 ~ (R1AA * lnB0 + log2(R1BB))

Parameters:

Estimate Std. Error t value Pr(>|t|)

R1AA -0.273817 0.025137 -10.89 8.36e-15 ***

R1BB 0.940178 0.009764 96.29 < 2e-16 ***

---

Signif. codes: 0 ‘***’ 0.001 ‘**’ 0.01 ‘*’ 0.05 ‘.’ 0.1 ‘ ’ 1

Residual standard error: 1.944 on 50 degrees of freedom

Correlation of Parameter Estimates:

R1AA

R1BB -0.40

Number of iterations to convergence: 2

Achieved convergence tolerance: 2.917e-05

> model1e <- nls(lnR1 ~ (R1AA*lnB0+log2(R1BB)), data=R1lungs, start=list(R1AA=-0.2, R1BB=1), weights=N_sp_w, control=list(maxiter = 500, tol = 0.001, minFactor = 1e-7, printEval = TRUE, warnOnly = FALSE))

It. 1, fac= 1, eval (no.,total): ( 1, 1): new dev = 9.46577

It. 2, fac= 1, eval (no.,total): ( 1, 2): new dev = 9.4649

> temp<-summary(model1e,100); print(temp)

Formula: lnR1 ~ (R1AA * lnB0 + log2(R1BB))

Parameters:

Estimate Std. Error t value Pr(>|t|)

R1AA -0.22841 0.04534 -5.037 5.82e-06 ***

R1BB 0.95839 0.01712 55.969 < 2e-16 ***

---

Signif. codes: 0 ‘***’ 0.001 ‘**’ 0.01 ‘*’ 0.05 ‘.’ 0.1 ‘ ’ 1

Residual standard error: 0.4226 on 53 degrees of freedom

Correlation of Parameter Estimates:

R1AA

R1BB -0.67

Number of iterations to convergence: 2

Achieved convergence tolerance: 4.453e-06

> model1f <- nls(lnR1 ~ (R1AA*lnB0+log2(R1BB)), data=R1lungs, start=list(R1AA=-0.2, R1BB=1), weights=unw_sp, control=list(maxiter = 500, tol = 0.001, minFactor = 1e-7, printEval = TRUE, warnOnly = FALSE))

It. 1, fac= 1, eval (no.,total): ( 1, 1): new dev = 1.06083

It. 2, fac= 1, eval (no.,total): ( 1, 2): new dev = 1.06061

> temp<-summary(model1f,100); print(temp)

Formula: lnR1 ~ (R1AA * lnB0 + log2(R1BB))

Parameters:

Estimate Std. Error t value Pr(>|t|)

R1AA -0.24008 0.04041 -5.941 2.25e-07 ***

R1BB 0.95006 0.01574 60.342 < 2e-16 ***

---

Signif. codes: 0 ‘***’ 0.001 ‘**’ 0.01 ‘*’ 0.05 ‘.’ 0.1 ‘ ’ 1

Residual standard error: 0.1415 on 53 degrees of freedom

Correlation of Parameter Estimates:

R1AA

R1BB -0.60

Number of iterations to convergence: 2

Achieved convergence tolerance: 9.582e-06

> model1g <- nls(lnR1 ~ (R1AA*lnB0+log2(R1BB)), data=R1lungs, start=list(R1AA=-0.2, R1BB=1), weights=var_ln_sp_notGRE_w, control=list(maxiter = 500, tol = 0.001, minFactor = 1e-7, printEval = TRUE, warnOnly = FALSE))

It. 1, fac= 1, eval (no.,total): ( 1, 1): new dev = 165.951

It. 2, fac= 1, eval (no.,total): ( 1, 2): new dev = 165.818

> temp<-summary(model1g,100); print(temp)

Formula: lnR1 ~ (R1AA * lnB0 + log2(R1BB))

Parameters:

Estimate Std. Error t value Pr(>|t|)

R1AA -0.22015 0.06636 -3.317 0.00239 **

R1BB 0.93044 0.02116 43.981 < 2e-16 ***

---

Signif. codes: 0 ‘***’ 0.001 ‘**’ 0.01 ‘*’ 0.05 ‘.’ 0.1 ‘ ’ 1

Residual standard error: 2.351 on 30 degrees of freedom

Correlation of Parameter Estimates:

R1AA

R1BB -0.63

Number of iterations to convergence: 2

Achieved convergence tolerance: 3.85e-05

> AIC(model1a,model2)

df AIC

model1a 3 -26.31355

model2 4 -31.28649

> AIC(model1a,model1b,model1c,model1d,model1e,model1f,model1g)

df AIC

model1a 3 -26.31355

model1b 3 -42.96368

model1c 3 -58.22758

model1d 3 -49.87091

model1e 3 -46.64501

model1f 3 -55.08342

model1g 3 -16.71254

Supplementary Material 3: Repeatability of ln(R_1_)

Table S2. Repeatability of $\ln(R_{1})$

| Study reference | B_0_ / T | N | Repeatability variance $\sigma_{L,wsws}^{2}$ |
| --- | --- | --- | --- |
| Tibiletti 2022 | 1.5 | 9 | 0.00283 |
| Alamidi 2016 | 1.5 | 27 | 0.00077 |
| Gai 2017 | 3.0 | 3 | 0.00004 |
| Neemuchwala 2020 | 1.5 | 5 | 0.05856 |
| Renne 2015 | 1.5 | 12 | 0.00034 |
|  |  |  |  |
| Weighted mean (1) |  | 56 | 0.00613 |
| Weighted mean (2) |  | 51 | 0.00099 |

1. N-weighted
2. N-weighted, omitting one outlying study
